# The Prevalence of Occult Celiac Disease among Patients with Functional Dyspepsia: A Study from the Western Region of Iran

**DOI:** 10.1155/2010/170702

**Published:** 2010-12-01

**Authors:** Ali Asghar Keshavarz, Homayoon Bashiri, Alireza Ahmadi, Shahrzad Bazargan-Hejazi

**Affiliations:** ^1^Department of Gastroenterology and Hepatology, Imam Reza Hospital, Kermanshah University of Medical Sciences (KUMS), Kermanshah, Iran; ^2^Department of Anesthesiology, Critical Care, and Pain Management, Kermanshah University of Medical Sciences (KUMS), Iran; ^3^Department of Public Health Sciences, Division of Social Medicine, Karolinska Institute, Stockholm, Sweden; ^4^Department of Psychology, College of Medicine, Charles Drew University of Medicine and Science, UCLA, Los Angeles, CA, USA; ^5^Psychiatry and Biobehavioral Sciences, Semel Institute, UCLA, Los Angeles, CA 90095, USA

## Abstract

*Objective*. The prevalence of Celiac Disease (CD) is high in Iran, and evaluation of CD is not part of the routine screening procedure for dyspeptic patients; therefore, cases of occult CD may be missed. This study aimed to investigate the prevalence of occult CD among dyspeptic patients who presented at a gastroenterology clinic in the Western region of Iran. *Methods*. In this descriptive, cross-sectional prospective study, patients who had a history of at least 12 weeks of upper abdominal discomfort were eligible to participate in the study during a 14-month recruitment period. Patients with a clinical or paraclinical data in favor of organic causes were excluded from the study. Enrolled patients were screened for IgA antiendomysium antibody (EMA) and IgA antitissue transglutaminase antibody (tTG). Those who screened positive for EMA/tTG received a confirmatory diagnostic biopsy for Marsh classification of CD. *Results*. From 225 potential participants with dyspepsia, 55 patients were excluded due to having explainable organic causes. The study sample included 170 patients with “functional dyspepsia.” Mean age of participants was 31 years and 55.8% were female. Twelve patients (7%) had positive tests (EMA/tTG), of which 10 were female (83.4%). According to Rome II criteria, all twelve patients with positive tests had “dysmotility type dyspepsia.” Based on Marsh classification, six patients were consistent with “Marsh I,” four with “Marsh II,” and two with the “Marsh III” classification. *Conclusions*. In this study, the prevalence of CD in dyspeptic patients was high. As a result, this study suggests that screening by serology tests (EMA/tTG) is justifiable for the detection of CD among functional dyspeptic patients in the tertiary centers in our country.

## 1. Introduction

Celiac disease (CD) is a genetic, inflammatory digestive disease triggered by ingestion of gluten-containing foods, which damage the small intestine and interfere with the absorption of nutrients from food [1]. CD has protean manifestations almost all of which are secondary to nutrient malabsorption and causes symptoms throughout the body, including abdominal bloating and pain, chronic diarrhea, vomiting, constipation, weight loss, and development of gastrointestinal cancer. It is prevalent in both adults and children, but is more frequent in women than men with F/M ratio 2 : 1 [2]. In the past, CD was thought to be a European disease, but recently, in developing countries, including the Middle East, Gluten intolerance CD has become a widespread public health problem in both the general population and in at-risk groups [3–5].

Nevertheless, nowadays only a small percentage of CD patients present with classic malabsorption syndrome. Many of these patients are oligosymptomatic, and usually present with a mild or silent form of the disease; a phenomenon called “celiac iceberg” by researchers. “Celiac iceberg” represents people at risk for celiac disease by virtue of their genes [3]. In the Middle East, the prevalence of the silent form in the general population has been estimated between 0.6%–1% [5, 6].

Since the first report of its atypical presentation [7], many investigators have tried to recognize its presentation within other intestinal and extraintestinal disorders [3, 8, 9]. Unrecognized cases of CD have the potential to cause severe complications, such as ulcerative, jejunoileitis malignancy, and autoimmune disease [8–10]. Early detection of this disease can prevent many of its unwanted complications. Therefore, efforts to recognize the silent or atypical forms of CD among high-risk groups should continue. The prevalence of CD among high-risk adult Iranian patients (i.e., diabetes mellitus type 1) who were screened for its subclinical presentation was reported between 2.4%–10% [11]. Among a sample of 2000 healthy Iranian blood donors, the prevalence of gluten sensitivity was estimated at 0.6% [5]. In a report from Turkey, among children with short stature, 55.3% screened positive for the silent form of CD [10]. Studies with regard to dyspeptic patients have been limited [12, 13]. In a study that evaluated the prevalence of CD in dyspeptic patients, including both functional and organic causes, researchers found a prevalence of 1.4% [12]. Dyspepsia is a common disease and affects about 40% of the general population. Even after its diagnosis, the cause of 60%–70% of these cases cannot be determined, and are generally labeled “functional dyspepsia” [12–17]. Pathological changes in the autonomous system have been suggested as the mechanism underlying the pathophysiology of functional dyspepsia subgroups. Antibody against enteric nervous system may play a role in functional symptoms seen in CD [18]. However, there are still many unresolved pathological aspects in the field of functional dyspepsia. Therefore, the specific aim of this study was to investigate the prevalence of occult form of CD among functional dyspeptic patients who presented to a gastroenterology clinic located in the Northwestern region of Iran. Findings from this study could help physicians to recognize the clinical presentations of celiac disease (i.e., classical, atypical and silent forms), and therefore pursue subsequent required referral and treatment.

## 2. Methods

In this prospective cross-sectional study, during a period of 14 months (October 2007 to December 2008), all patients with dyspepsia who were referred to our referral gastroenterology clinic in Imam Reza hospital, an affiliate of Kermanshah University of Medical Sciences in the western region of Iran, were recruited for participation in the study. Inclusion criteria included history of at least 12 weeks of upper abdominal discomfort. Both genders were eligible to participate, and there were no inclusion criteria for age. During the recruitment period, the objective of the study was explained to the potential participants, and informed consent was obtained. Patients with a history of CD in their family and clinical or paraclinical data in favor of GERD, IBS, drug usage (NSAID), pancreatic or gallbladder disease were excluded from study. Total abdominal sonography was done by an expert radiologist. Endoscopy was performed via videoendoscope (Pentax-EG2930K, Japan). From 225 potential dyspeptic participants with Rome II criteria, 55 patients were excluded due to having explainable gastrointestinal organic causes (Table 1). One hundred and seventy (170) patients who had no explainable organic cause with possible diagnosis of functional dyspepsia were enrolled in the study and classified into three subtypes according to Rome II criteria: (1) ulcer-like dyspepsia, where the predominant symptom is pain centered in the upper abdomen; (2) dysmotility-like dyspepsia, characterized by upper abdominal fullness, early satiety, bloating, or nausea, with no report of painful discomfort in the center or upper abdomen; (3) unspecified dyspepsia, where symptoms do not fulfill the criteria for ulcer-like or dysmotility-like dyspepsia. All three groups were screened for IgA antiendomysium antibody (EMA) and IgA antitissue transglutaminase antibody (tTG), which were detected by the ELISA test (BMD, Marne la Vallee, France). In patients with positive EMA/tTG, titer (>20 iu/mL), re-endoscopy was performed and at least four biopsy fragments from the distal portion of the duodenum were taken, fixed in 10% formaldehyde, and sent to the pathology lab. The pathologist was unaware of the clinical or endoscopy diagnosis of the patients. The small intestinal biopsy is considered the gold standard for diagnosis of CD using the modified Marsh UEGW criteria [14] for the analysis of fragment. Descriptive statistics were performed using SPSS for Windows (version12).

## 3. Results

Table 1 presents the organic causes for the enrolled patients who were excluded from study.  Table 2 presents the demographic characteristics and prevalence of CD among the functional dyspeptic subtypes. A majority of the enrolled sample were female (55.8%). Positive EMA/tTG was reported among 7% of the sample (*n* = 12 patients), of whom 10 were female (83.4%). All of the patients with EMA/tTG (*n* = 12) had “Dysmotility type dyspepsia,” according to the Rome II subclassifications. Based on Marsh classification, six of these patients were consistent with “Marsh I,” four were with “Marsh II,” and two with the “Marsh III” classification.

## 4. Discussion

CD is a curable disease; however, it poses a challenging public health problem in developing countries [3]. Previous studies have reported high prevalence of CD among patients with organic or functional dyspepsia [13, 14], but to our knowledge, this is the first study that reports the prevalence of CD in dyspeptic patients according to Rome II subclassification. Over the last decade, clinical researchers have revealed that CD is no longer a rare disease, and its subclinical or atypical form is quite prevalent [3]. Therefore, it has been recommended to detect the symptoms of this curable disease in its initial presentation in order to prevent its unwanted complications. Three approaches have been suggested for CD screening [3]. One option is to perform a biopsy on all patients who undergo upper endoscopy [2, 17]. This approach has been criticized due to the costly nature of this procedure. The second approach suggests using magnification tools. However, this approach is less practical and costly for screening. The third approach offers the serology tests (EMA/tTG). These tests, regardless of their limitations, seem to be the most cost-effective approach available for screening CD in the general population or in high-risk groups. In this study, the prevalence of dyspepsia using EMA/tTG was 7%, a figure higher than what has been reported in the general population [3, 5]. Furthermore, all patients with positive serology or histopathology in favor of CD had the Dysmotility type functional dyspepsia according to Rome II criteria. The pathophysiology of the Dysmotility type dyspepsia has been attributed to autoimmune damage to autonomous system or increase in neurotensin or enteroglucagon levels which impair gastrointestinal motility [15, 16, 18]. These pathological changes may be the mechanism that explains the higher prevalence of CD in this subtype group of dyspeptic patients. The higher prevalence of CD among females in our sample is in agreement with other studies, which have shown similar results among women [3, 12, 13]. The rationale for the latter finding has not been clarified in the literature. In our study, more than 50% of the patients were in the Marsh stage I, suggesting that diagnosing patients in an early stage can lead to proper management and prevention of late and severe complications. The reasons as to why most of the patients in this study were in the Marsh I stage is not clear, but to our knowledge the atypical pictures of CD in contrast to its classical forms may have less pathologic changes of gluten enteropathy in the gut.

This study identifies a high prevalence of CD in functional dyspeptic patients (7%) using diagnostic biopsy from the second portion of the duodenum. The prevalence of CD was higher among female patients (83.4%) and Dysmotility type dyspepsia (50%). These findings suggest that performing a routine diagnostic biopsy among at-risk patients (i.e., functional dyspeptic patients and female patients with dominant Dysmotility type functional dyspepsia) can identify CD in its earliest stage and delay more complicated life threatening and terminal complications. In developing countries, early screening and identification of CD in at-risk groups can increase the level of awareness and sensitivity of physicians in recognizing various clinical presentations of CD and allow them to treat patients accordingly.

## 5. Limitations and Future Directions

This study offers several strengths. Most prominently, it is among the first study that is designed to focus on occult celiac disease among patients with functional dyspepsia. Furthermore, the study was conducted in a geographic area with the higher rates of CD in the world. Despite these strengths our study is subject to several limitations. The sample size was small and was collected only in one region of Iran, hence limiting our ability to conduct more rigorous statistical tests on the data, as well as limiting our ability to generalize the study findings to a larger population in the country or other parts of the world. Future control trial studies with a larger sample and follow-up with gluten-free diet are needed to confirm and expand upon these findings.

## Figures and Tables

**Table 1:** Organic causes among the enrolled dyspeptic patients.

Diagnosis	*N* (%)
Peptic lesions	35 (64)
Esophagitis	8 (15)
Erosive gastritis	11 (20)
Duodenal ulcer	10 (18)
Gastric ulcer	6 (11)
Malignancy	7 (12)
Adenocarcinoma	5 (71.4)
Gastric lymphoma	2 (28.6)
Miscellaneous	13 (24)
Gastric polyp	4 (30.7)
Telangiectasia	2 (15.3)
Heterotopic pancreas	1 (7.69)
Leiomyoma	2 (15.3)
Portal gastropathy	1 (7.69)
Lymphocytic gastritis	2 (15.3)
Cystic fundal hyperplasia	1 (7.69)
Total	50 (100)

**Table 2:** Demographic data of patients with functional dyspepsia.

Mean age (yr) ±SD	31 ± 3.4
Age range (yr)	12–75
Female	95 (55.8%)
Male	75 (44.1%)
Ulcer-like	51 (30%)
Dysmotility	85 (50%)
Nonspecific	34 (20%)
Positive EMA/tTG	12 (7%)
